# Supplementary material for: Hiding in plain sight: Genomic and phenotypic characterization of mosquito-borne Bussuquara virus
Source: PLoS Negl Trop Dis. 2025 Dec 5;19(12):e0013774. doi: 10.1371/journal.pntd.0013774 (PMC12680234; doi:10.1371/journal.pntd.0013774)
Supplement: S1 File — (PDF) [file pntd.0013774.s001.pdf]

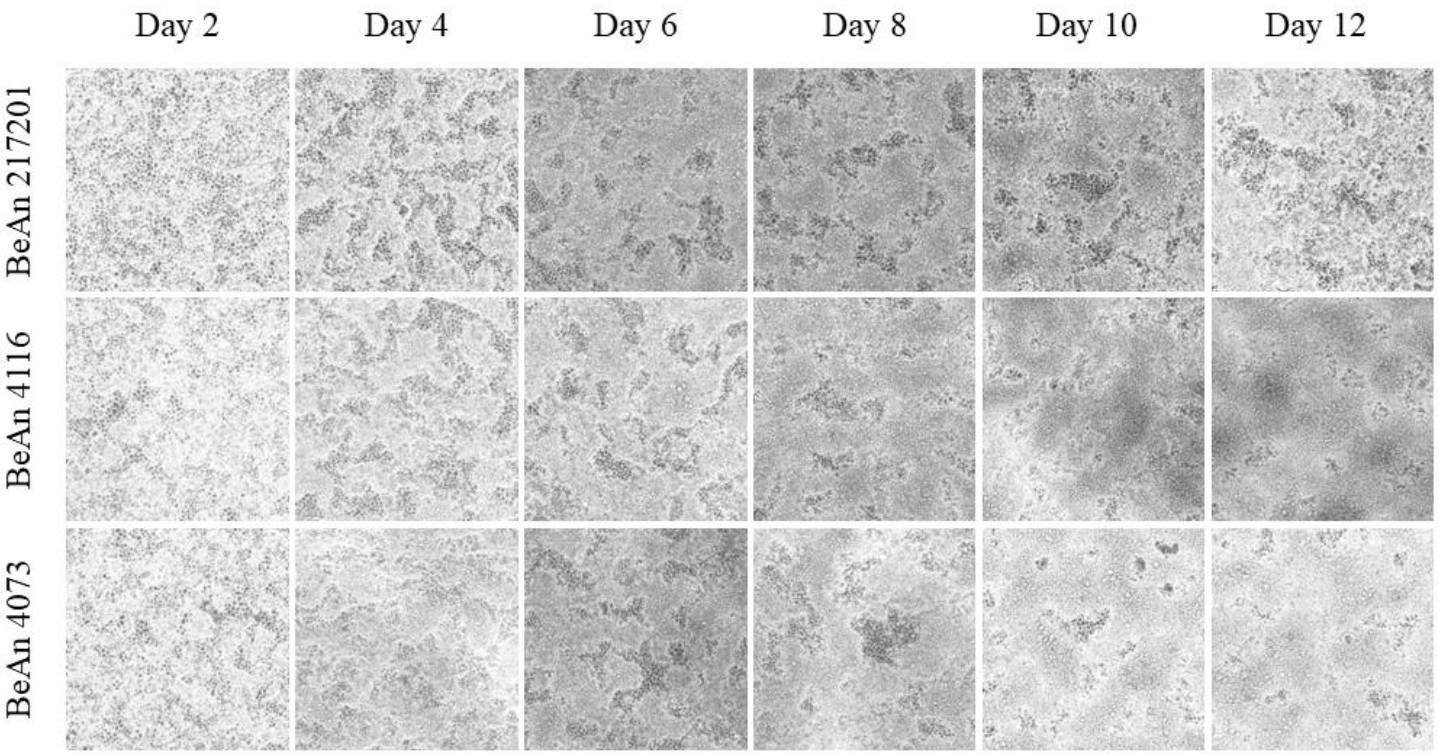


**Figure S1. Bi-daily monolayer documentation of C6/36 (*Aedes albopictus*) cells infected with BSQV**
C6/36 cells were infected with three strains of BSQV at a multiplicity of infection of 0.01. Monolayer integrity was documented every other day from days two to twelve post-infection using a light microscope under 10x magnification.

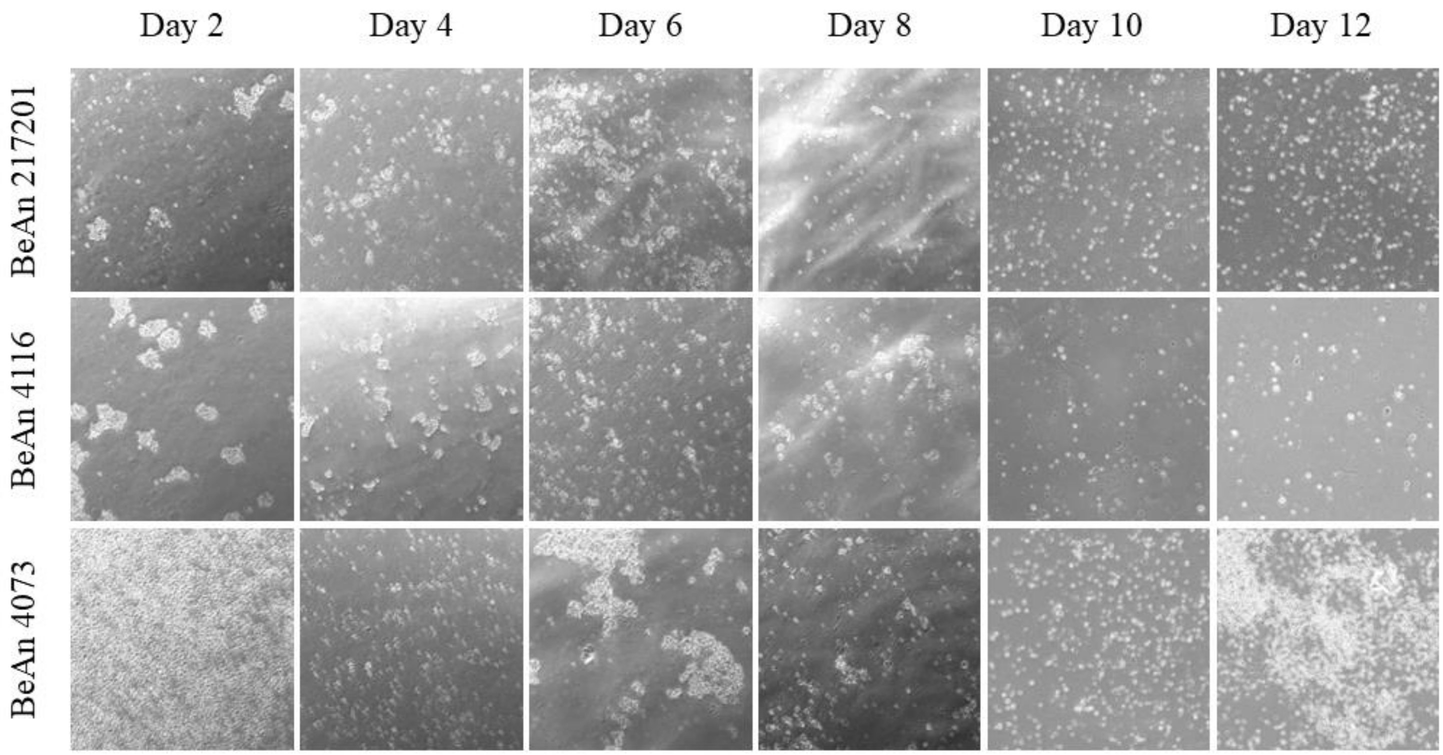


**Figure S2. Bi-daily monolayer documentation of U4.4 (*Aedes albopictus*) cells infected with BSQV**
U4.4 cells were infected with three strains of BSQV at a multiplicity of infection of 0.01. Monolayer integrity was documented every other day from days two to twelve post-infection using a light microscope under 10x magnification.

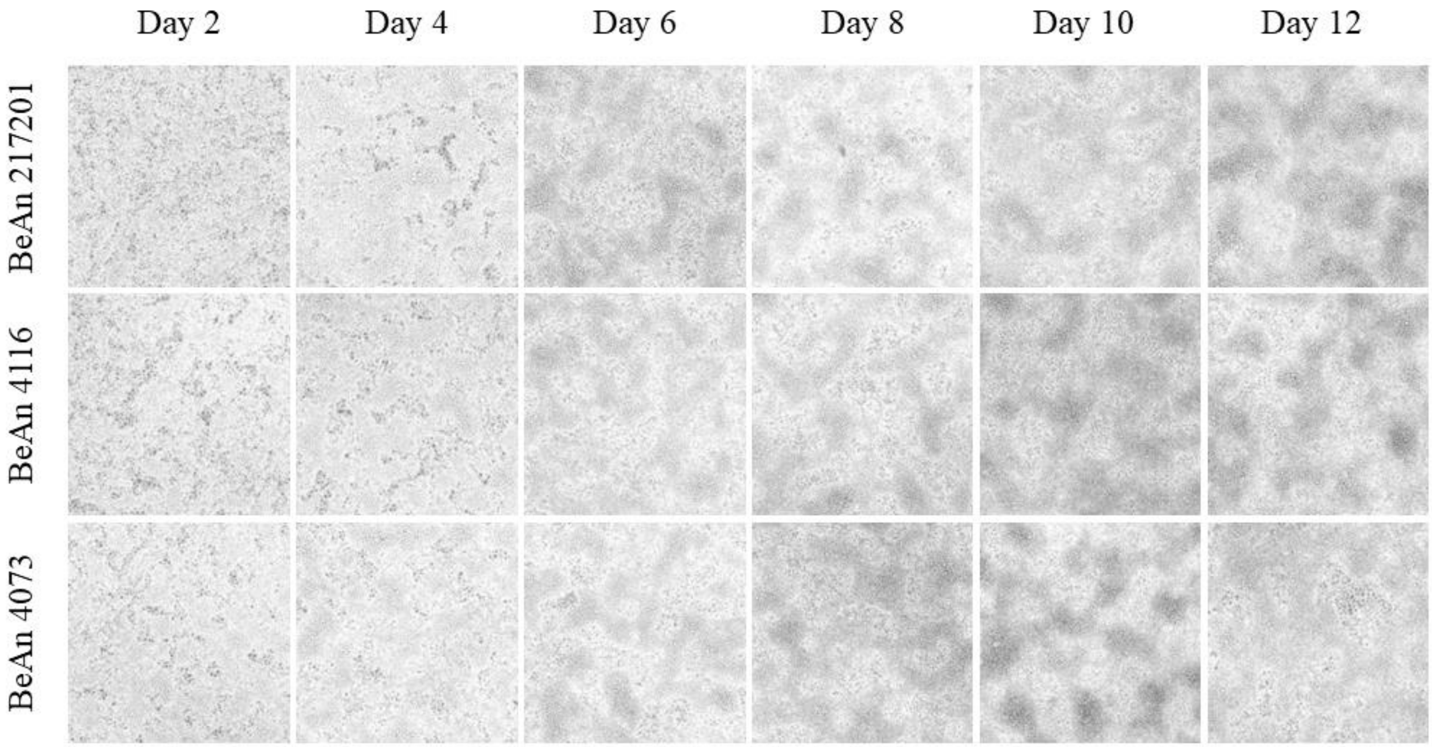


**Figure S3. Bi-daily monolayer documentation of C7-10 (*Aedes albopictus*) cells infected with BSQV**
C7-10 cells were infected with three strains of BSQV at a multiplicity of infection of 0.01. Monolayer integrity was documented every other day from days two to twelve post-infection using a light microscope under 10x magnification.

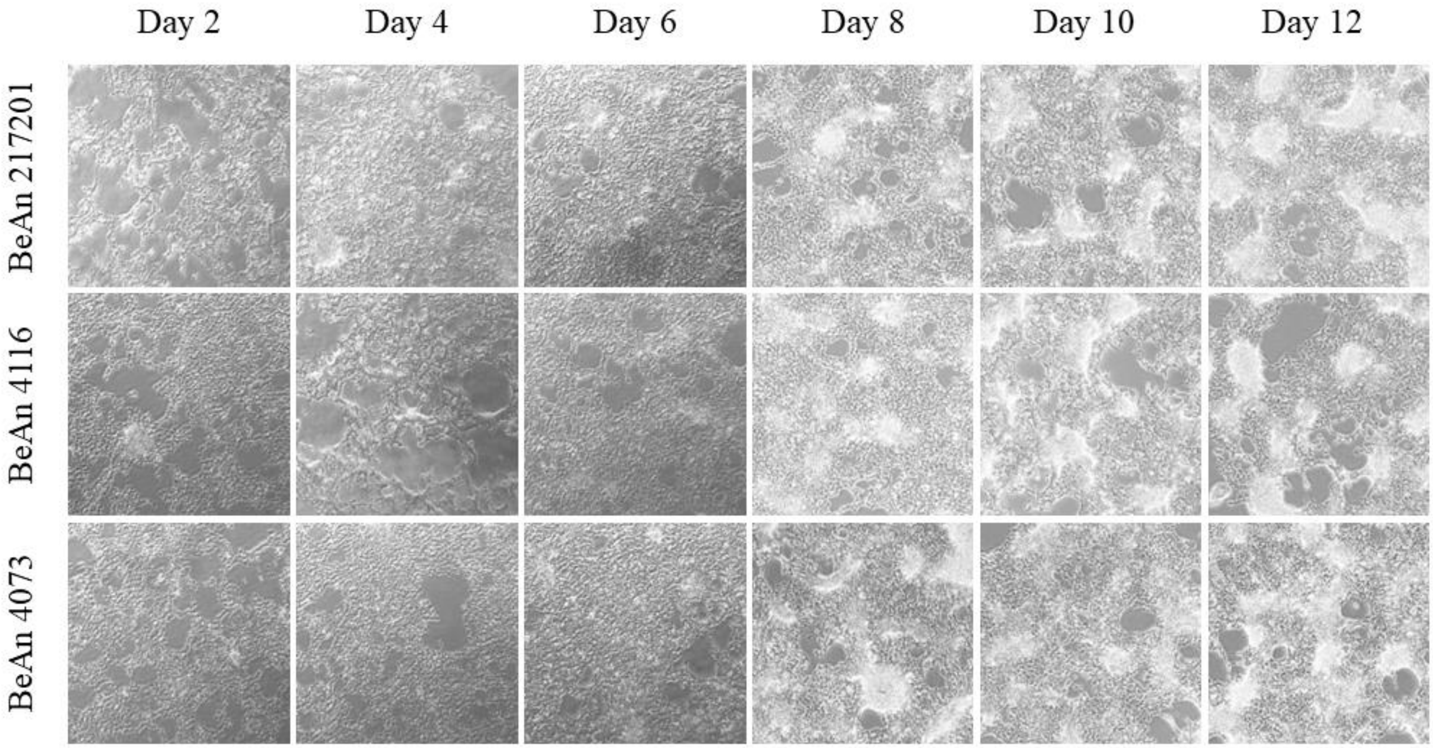


**Figure S4. Bi-daily monolayer documentation of Aag2 (*Aedes aegypti*) cells infected with BSQV**
Aag2 cells were infected with three strains of BSQV at a multiplicity of infection of 0.01. Monolayer integrity was documented every other day from days two to twelve post-infection using a light microscope under 10x magnification.

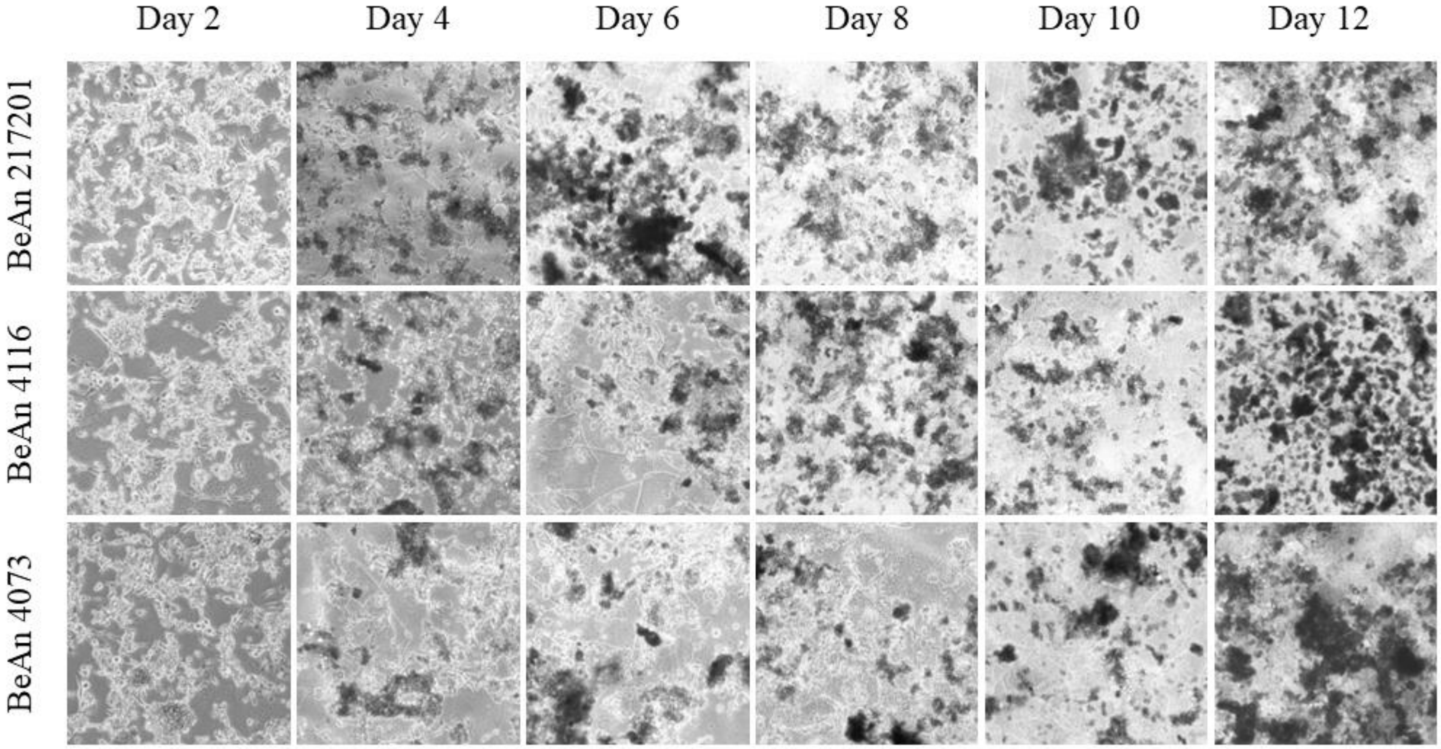


**Figure S5. Bi-daily monolayer documentation of CxTr (*Culex tarsalis*) cells infected with BSQV**
CxTr cells were infected with three strains of BSQV at a multiplicity of infection of 0.01. Monolayer integrity was documented every other day from days two to twelve post-infection using a light microscope under 10x magnification.

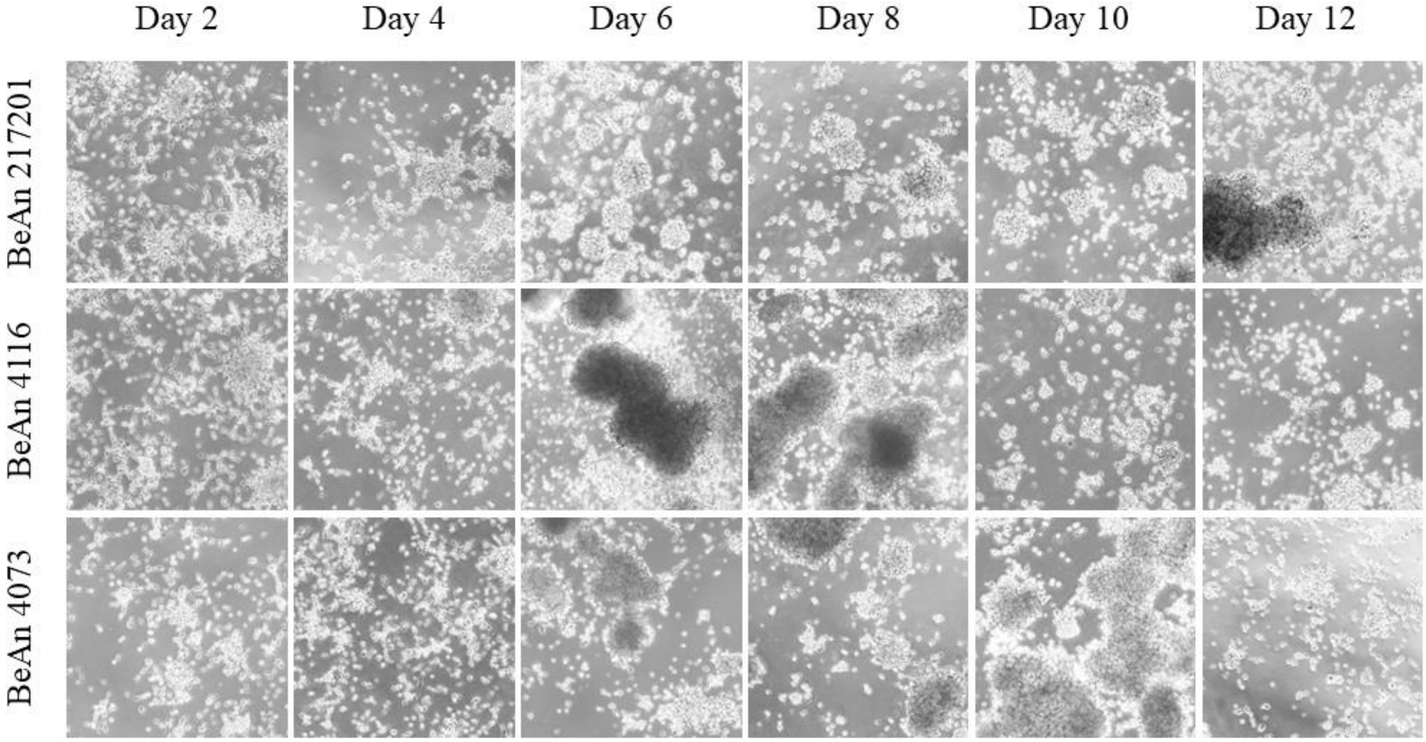


**Figure S6. Bi-daily monolayer documentation of CT (*Culex tarsalis*) cells infected with BSQV**
CT cells were infected with three strains of BSQV at a multiplicity of infection of 0.01. Monolayer integrity was documented every other day from days two to twelve post-infection using a light microscope under 10x magnification.

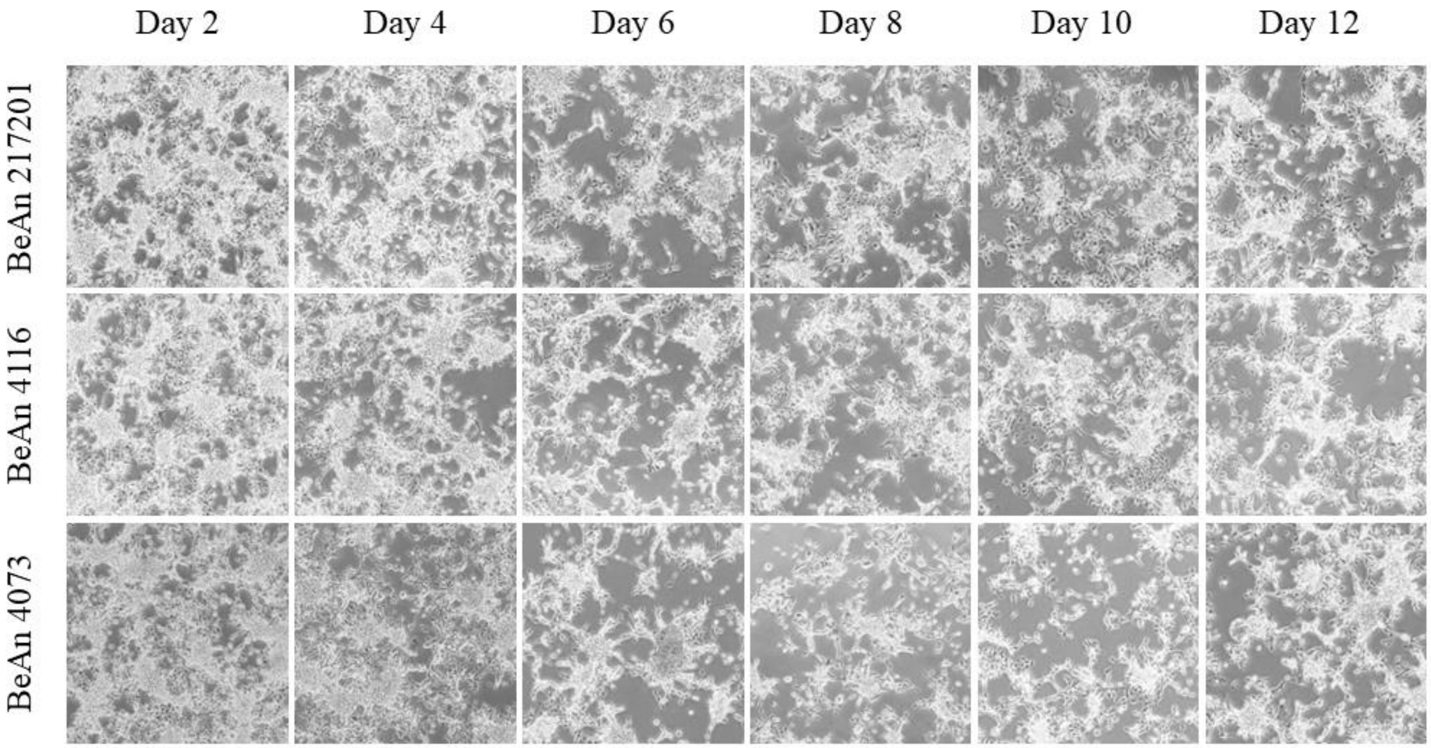


**Figure S7. Bi-daily monolayer documentation of Hsu (*Culex quinquifasciatus*) cells infected with BSQV**
Hsu cells were infected with three strains of BSQV at a multiplicity of infection of 0.01. Monolayer integrity was documented every other day from days two to twelve post-infection using a light microscope under 10x magnification.

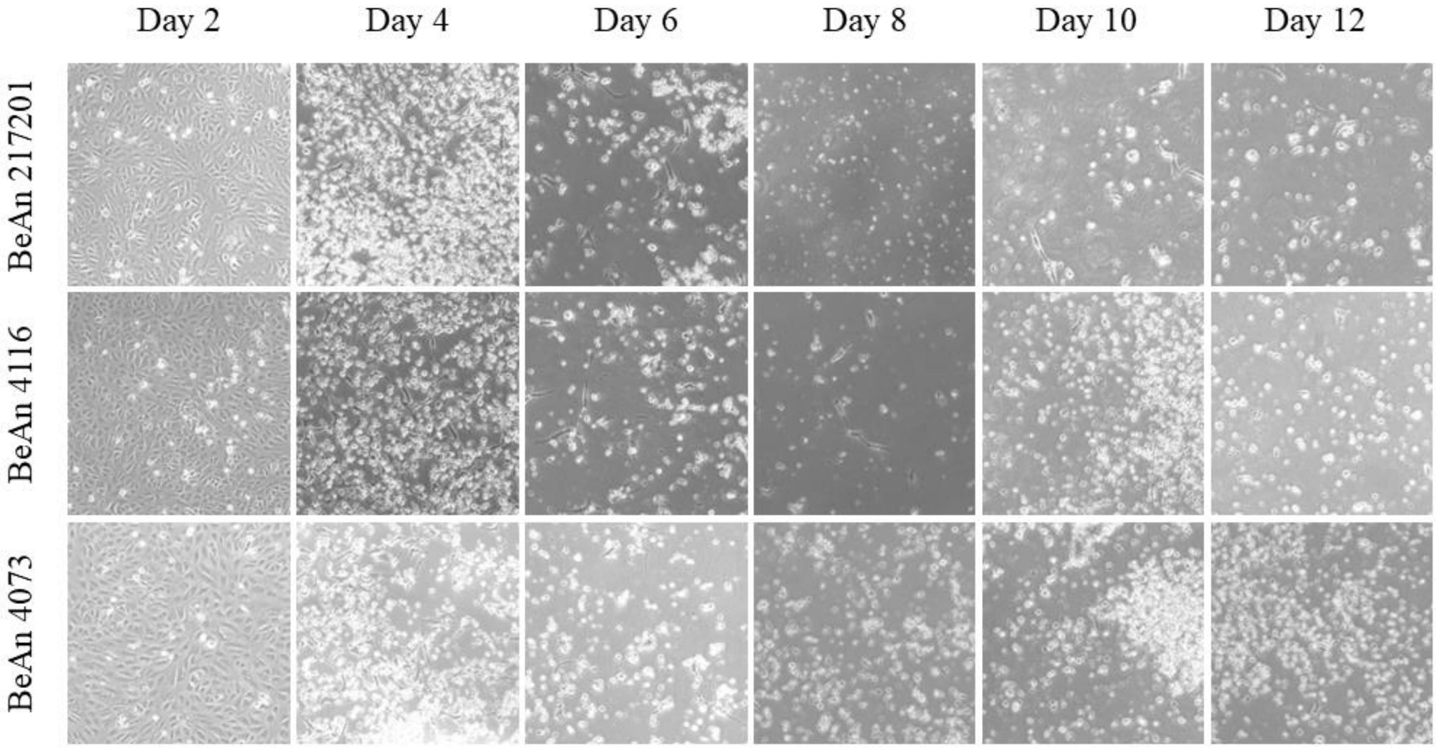


**Figure S8. Bi-daily monolayer documentation of Vero CCL81 (*Cercopithecus aethiops*) cells infected with BSQV**
Vero CCL81 cells were infected with three strains of BSQV at a multiplicity of infection of 0.01. Monolayer integrity was documented every other day from days two to twelve post-infection using a light microscope under 10x magnification.

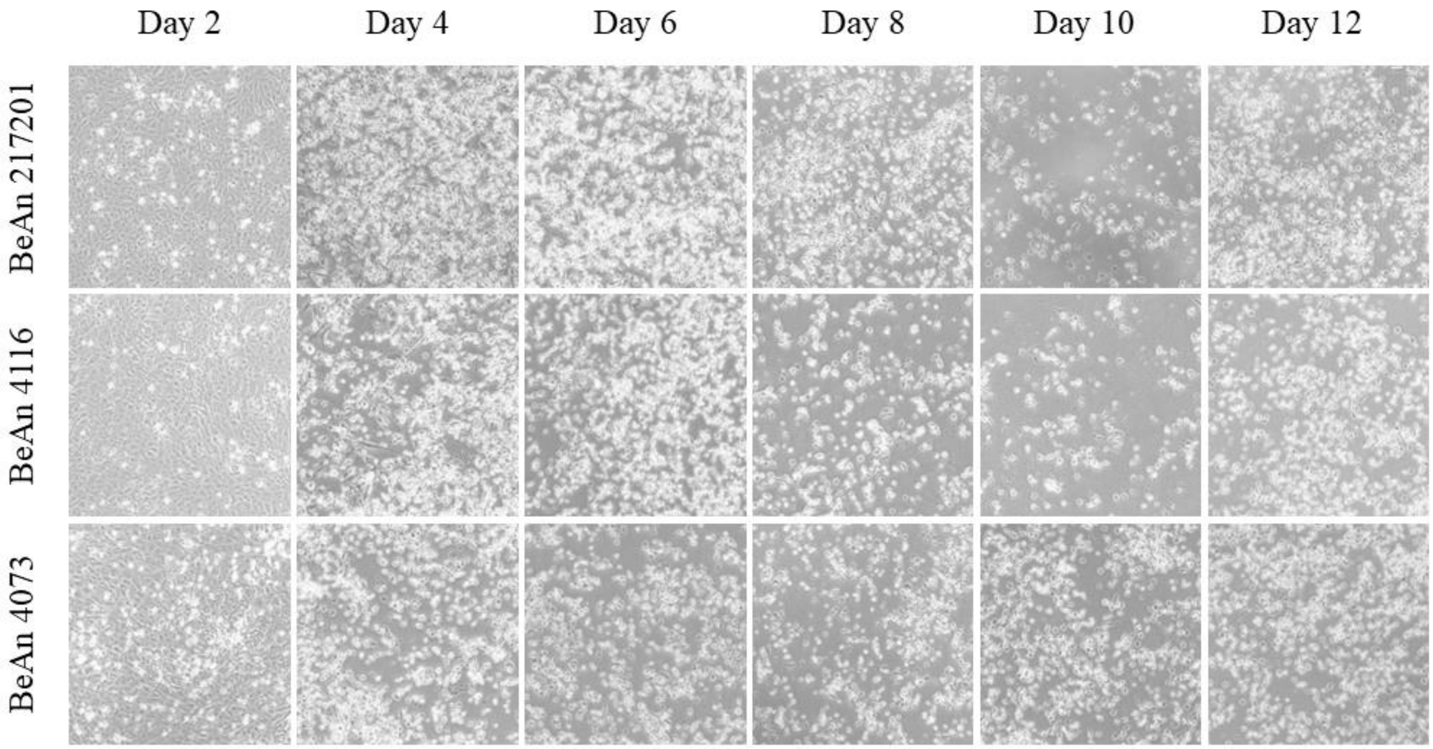


**Figure S9. Bi-daily monolayer documentation of OK (*Didelphis marsupialis virginiana*) cells infected with BSQV**

OK cells were infected with three strains of BSQV at a multiplicity of infection of 0.01. Monolayer integrity was documented every other day from days two to twelve post-infection using a light microscope under 10x magnification.

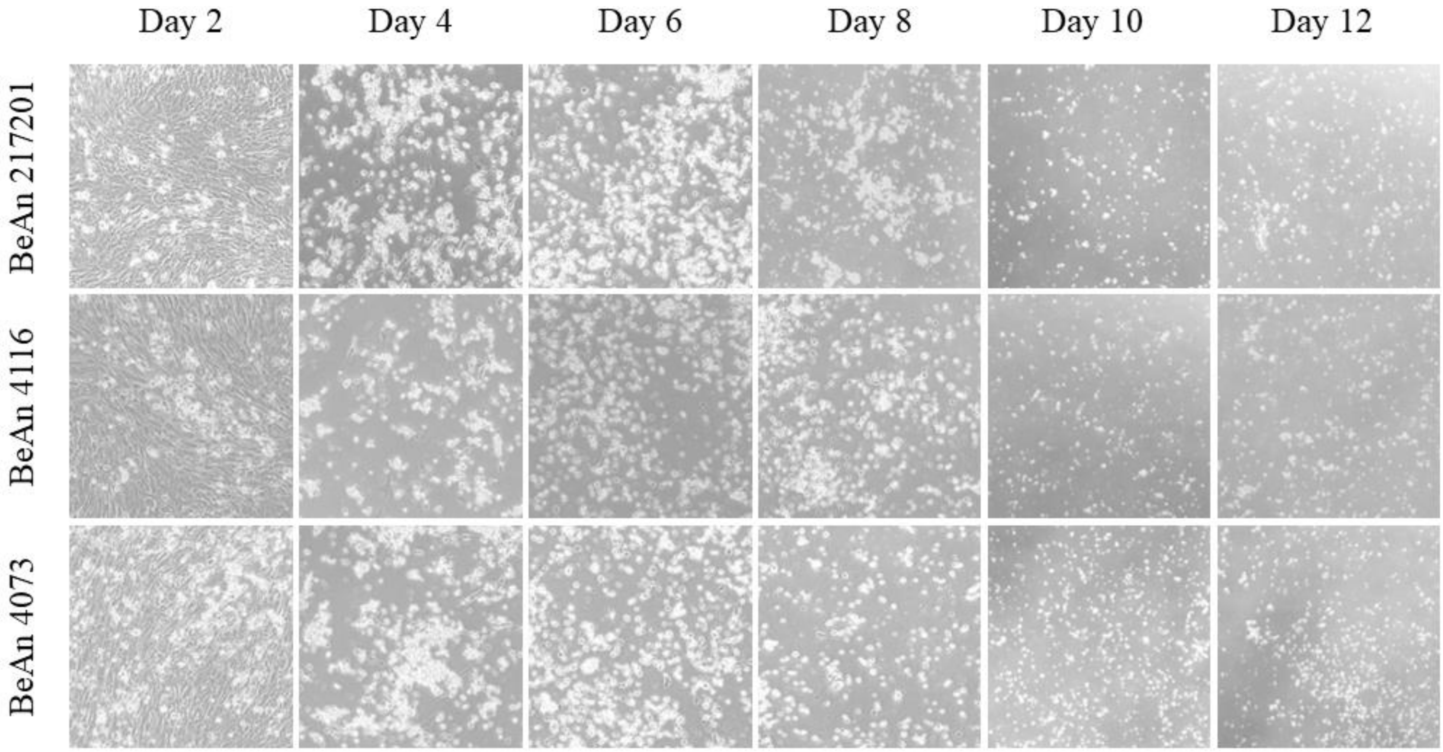


**Figure S10. Bi-daily monolayer documentation of BHK-21 (*Mesocricetus auratus*) cells infected with BSQV**

BHK-21 cells were infected with three strains of BSQV at a multiplicity of infection of 0.01. Monolayer integrity was documented every other day from days two to twelve post-infection using a light microscope under 10x magnification.

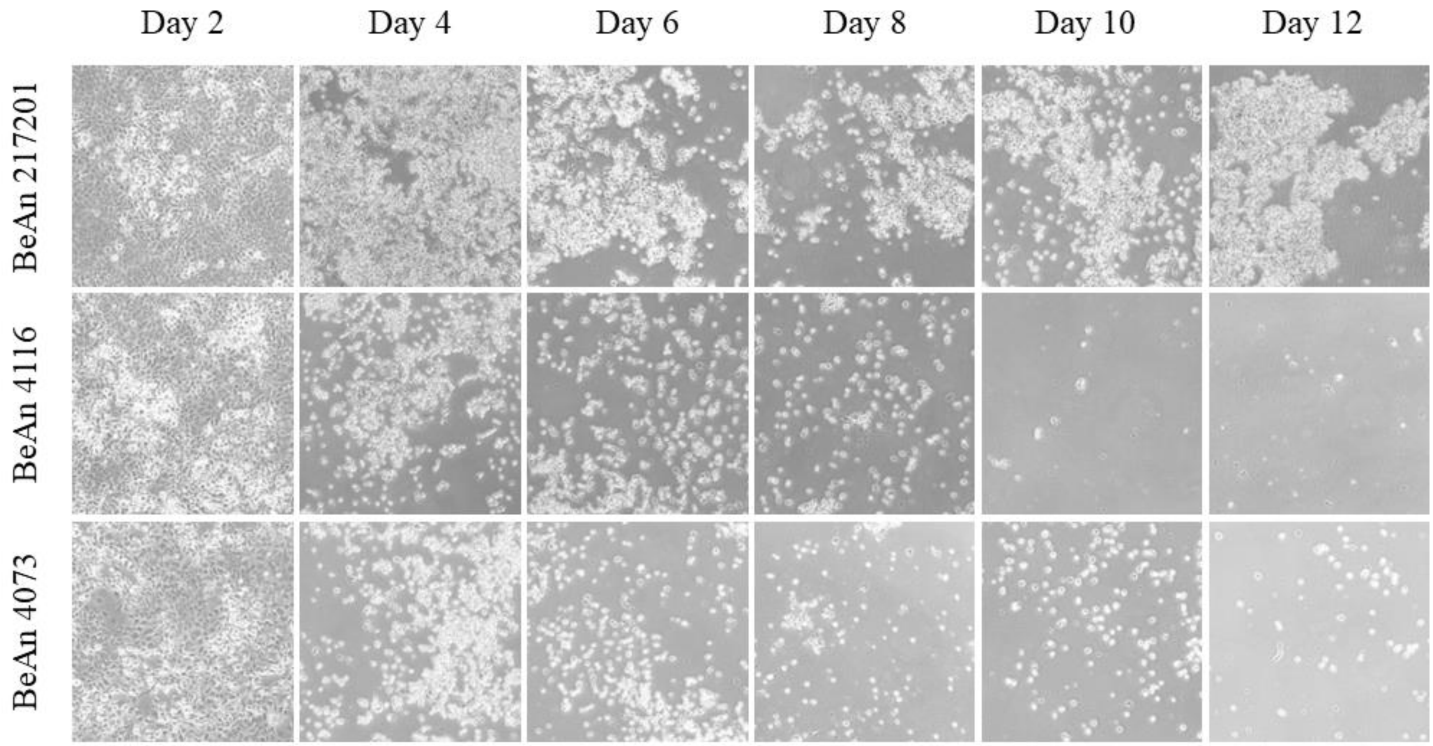


**Figure S11. Bi-daily monolayer documentation of Huh7 (*Homo sapiens*) cells infected with BSQV**
Huh7 cells were infected with three strains of BSQV at a multiplicity of infection of 0.01. Monolayer integrity was documented every other day from days two to twelve post-infection using a light microscope under 10x magnification.

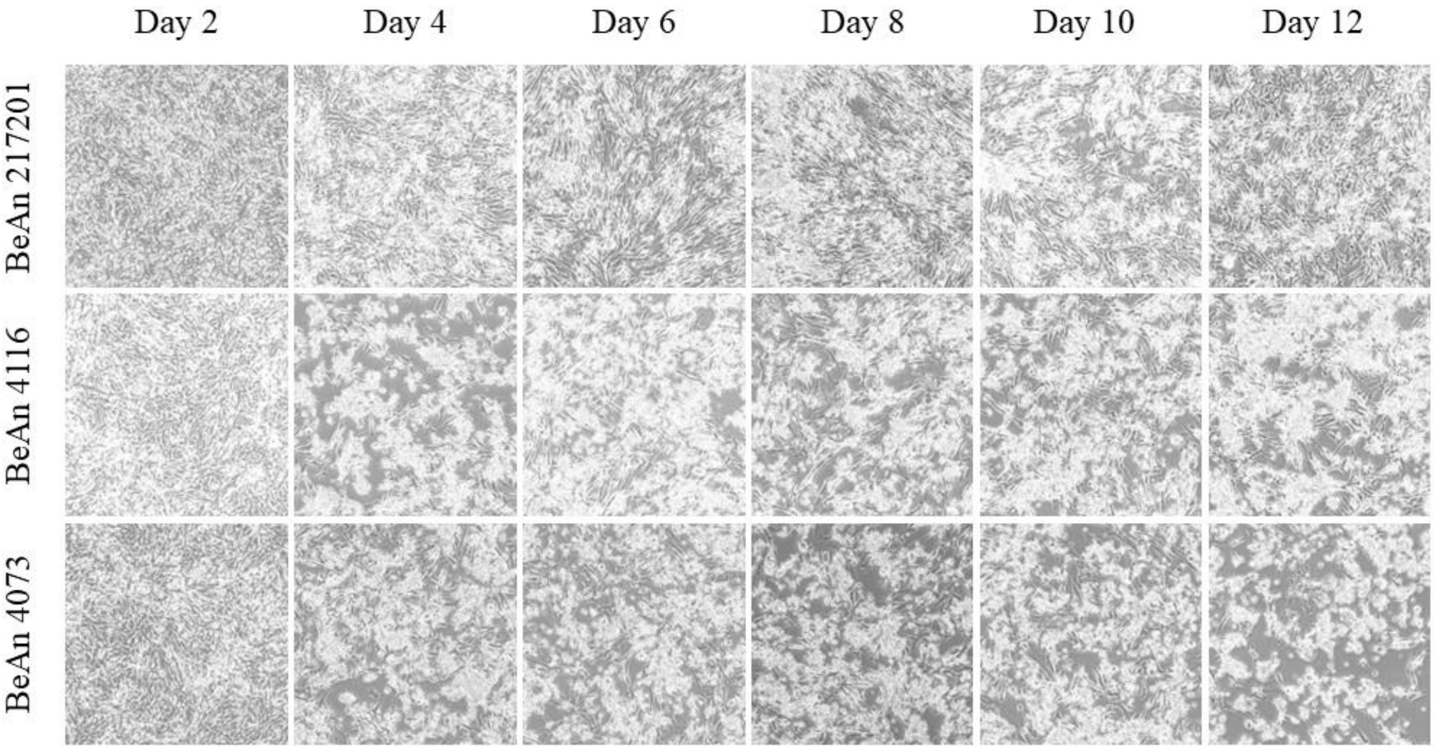


**Figure S12. Bi-daily monolayer documentation of QM7 (*Coturnix coturnix japonica*) cells infected with BSQV**
QM7 cells were infected with three strains of BSQV at a multiplicity of infection of 0.01. Monolayer integrity was documented every other day from days two to twelve post-infection using a light microscope under 10x magnification.

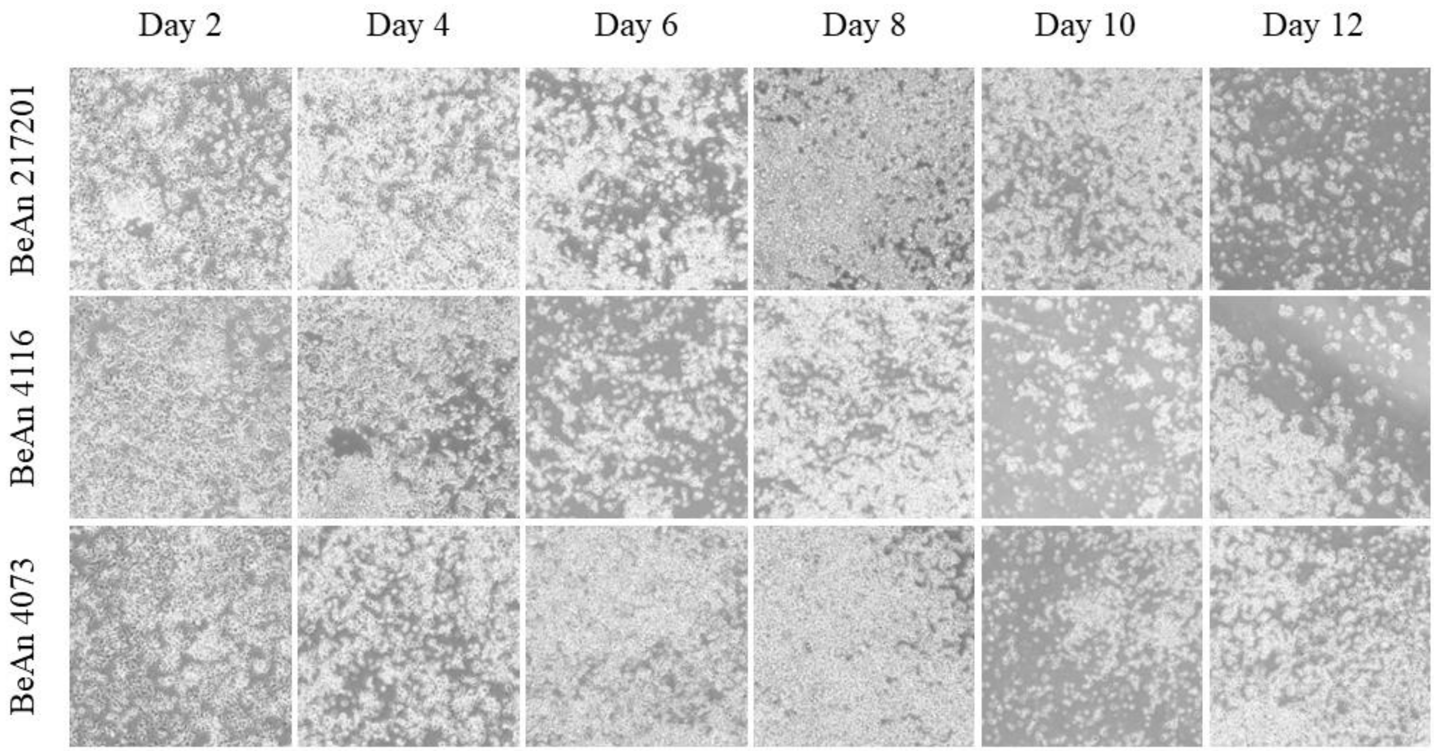


**Figure S13. Bi-daily monolayer documentation of LMH (*Gallus gallus*) cells infected with BSQV**
LMH cells were infected with three strains of BSQV at a multiplicity of infection of 0.01. Monolayer integrity was documented every other day from days two to twelve post-infection using a light microscope under 10x magnification.

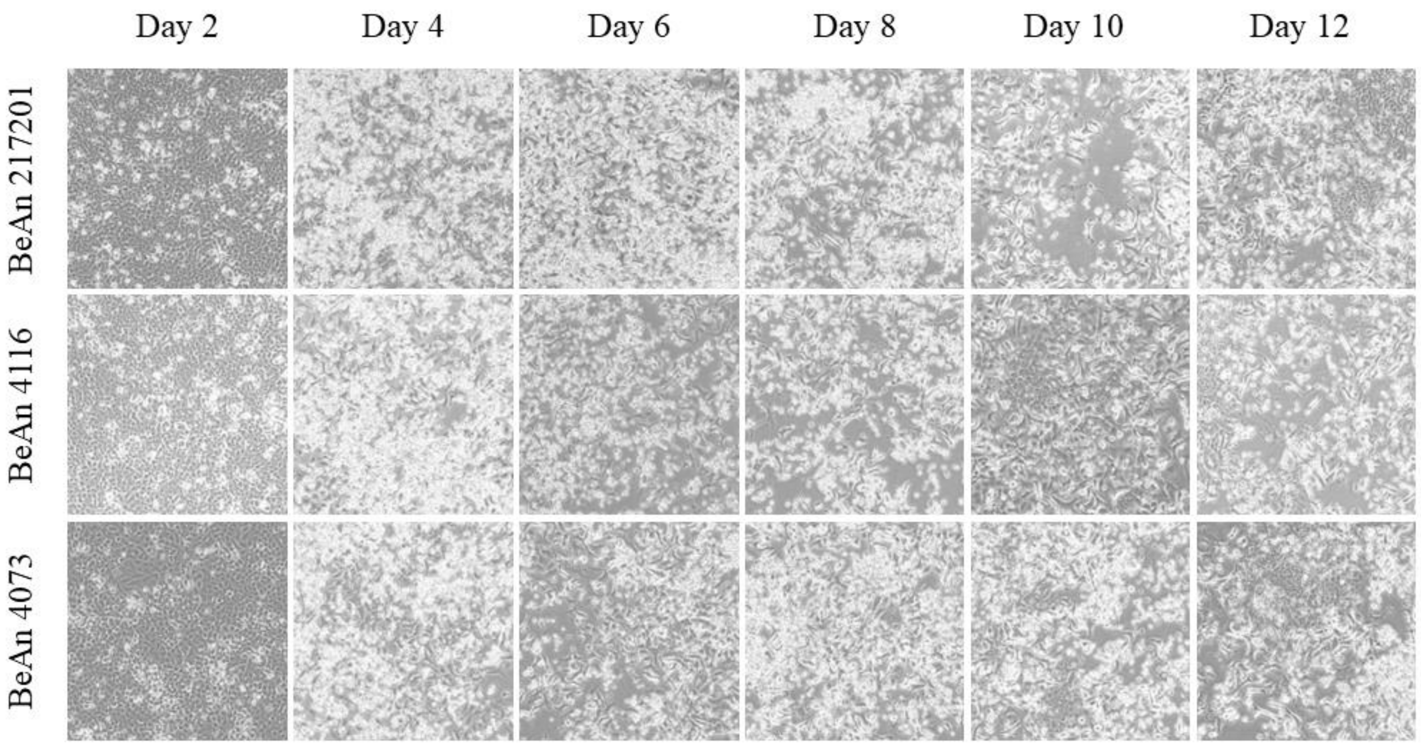


**Figure S14. Bi-daily monolayer documentation of A549 (*Homo sapiens*) cells infected with BSQV**
A549 cells were infected with three strains of BSQV at a multiplicity of infection of 0.01. Monolayer integrity was documented every other day from days two to twelve post-infection using a light microscope under 10x magnification.

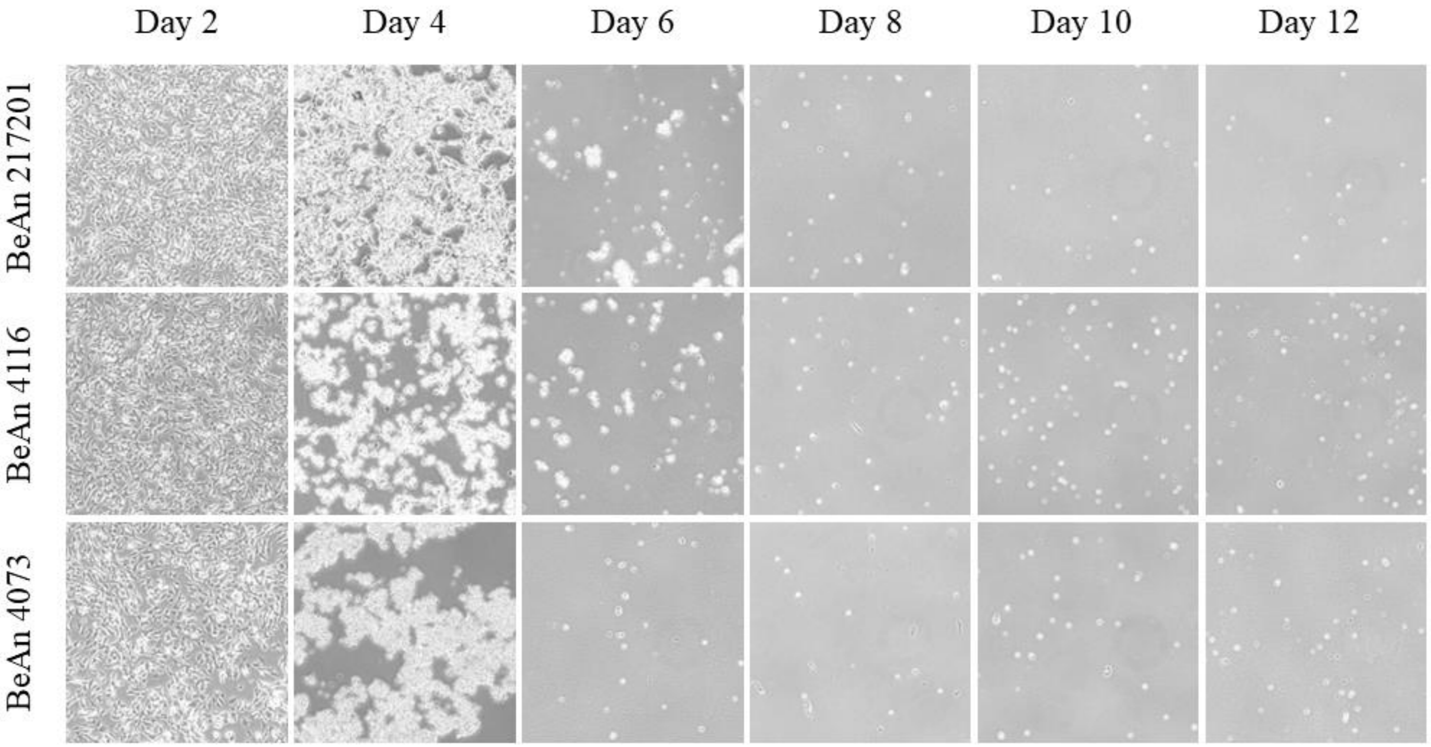


**Figure S15. Bi-daily monolayer documentation of SH-SY5Y (*Homo sapiens*) cells infected with BSQV**
SH-SY5Y cells were infected with three strains of BSQV at a multiplicity of infection of 0.01. Monolayer integrity was documented every other day from days two to twelve post-infection using a light microscope under 10x magnification.

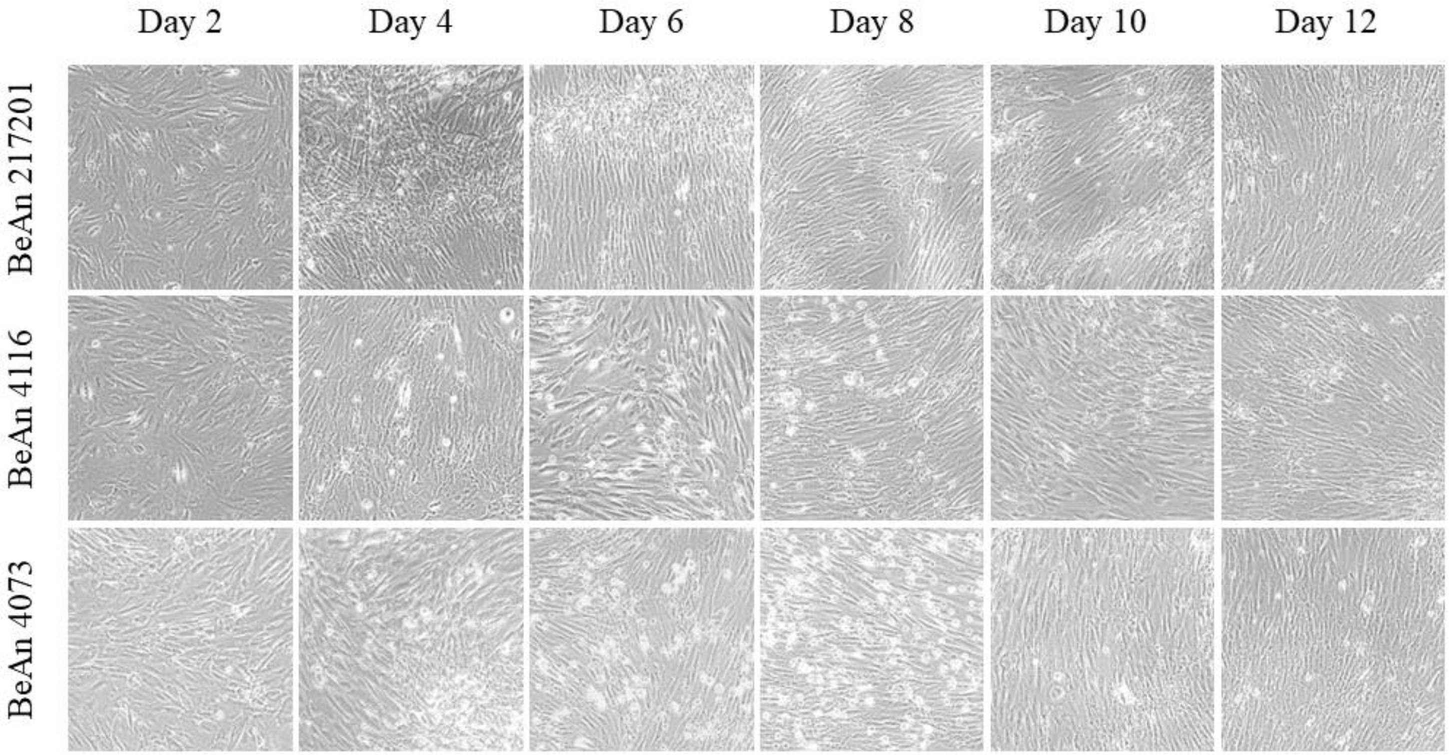


**Figure S16. Bi-daily monolayer documentation of FRhL-2 (*Macaca mulatta*) cells infected with BSQV**
FRhL-2 cells were infected with three strains of BSQV at a multiplicity of infection of 0.01. Monolayer integrity was documented every other day from days two to twelve post-infection using a light microscope under 10x magnification.

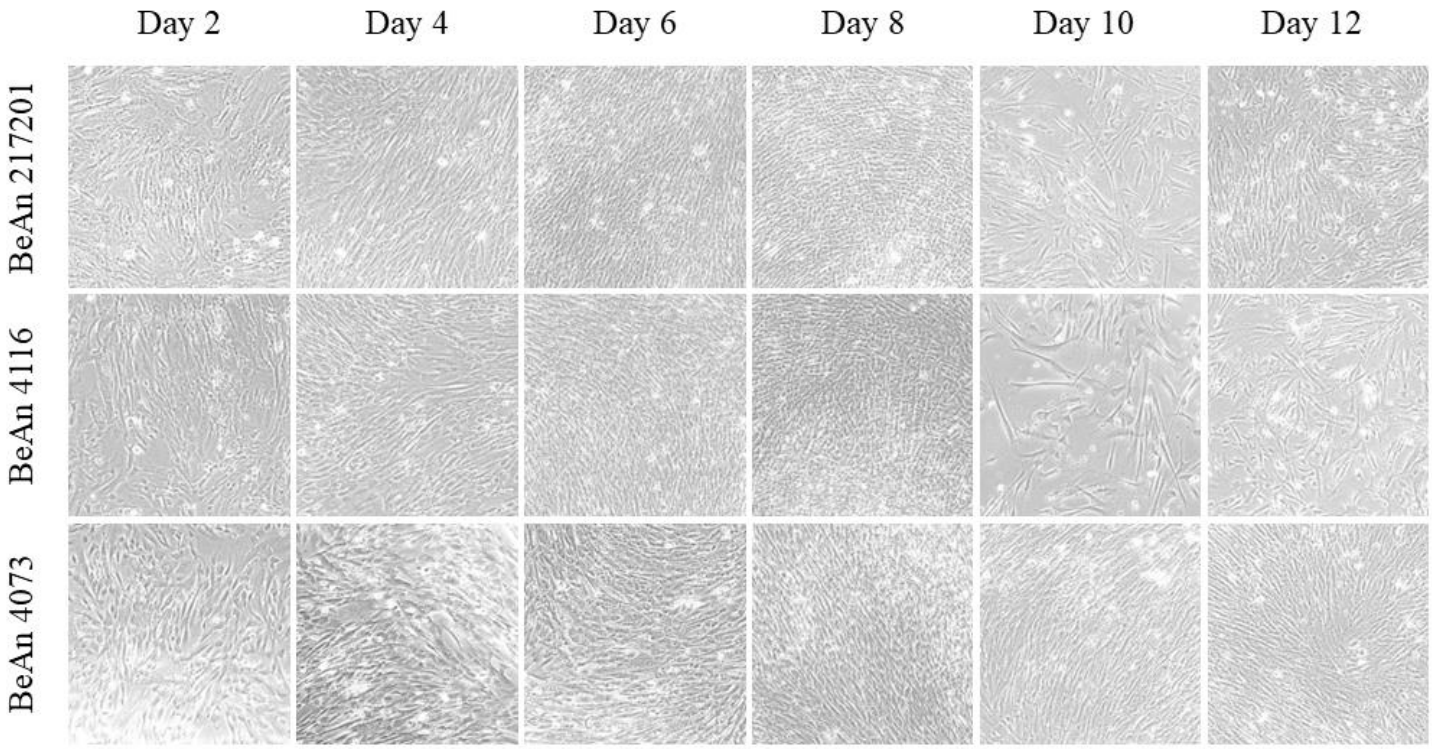


**Figure S17. Bi-daily monolayer documentation of HSerC (*Homo sapiens*) cells infected with BSQV**
HSerC cells were infected with three strains of BSQV at a multiplicity of infection of 0.01. Monolayer integrity was documented every other day from days two to twelve post-infection using a light microscope under 10x magnification.
