# Supplementary material for: Hiding in plain sight: Genomic and phenotypic characterization of mosquito-borne Bussuquara virus
Source: PLoS Negl Trop Dis. 2025 Dec 5;19(12):e0013774. doi: 10.1371/journal.pntd.0013774 (PMC12680234; doi:10.1371/journal.pntd.0013774)
Supplement: Attachment — Submitted filename: Response to Reviewers 10.09.2025.docx [file pntd.0013774.s008.docx]

**Responses to Reviewers' Comments**

**Reviewer #1**

**Methods:**
Reviewer #1: Yes the authors clearly stated their objectives and executed the studies in a rigorous manner.

Response: Thank you.

**Results:**
Reviewer #1: The results, figures and tables are clearly presented.

Response: Thank you.

**Conclusions:**
Reviewer #1: Yes, although I think the authors could have developed the conclusion/ discussion section more. Considering their observations that Bussuquara virus didn't replicate well in Aedes cell lines it would have been nice to see some mosquito infections. I know they have the facilities and mosquito colonies to test this.

Response: We modified the conclusion/discussion section to focus on the generalist arbovirus status of BSQV and downplay the emergence potential until vector competence (VC) experiments are performed. We would like to bring to the attention of the reviewer that VC was outside of the scope of this current manuscript, since we are coupling these experiments with understanding BSQV pathogenesis in mice currently under way and subject to a separate manuscript

**Editorial and Data Presentation Modifications?**
Reviewer #1: (No Response)

**Summary and General Comments:**

Reviewer #1: The authors characterized three strains of Bussuquara virus, an orthoflavivirus with potential epidemic potential, using genomic, phylogenetic, microscopic, and cell culture techniques. In general, their results demonstrate it is a stereotypical mosquito-borne orthoflavivirus. Interestingly, they did find that BSQV did not replicate in immune-competent Aedes cell lines while replicating in Culex cell lines just fine. It would have been nice to see some experimental mosquito work to complement their cell culture data. Further, the discussion section/ conclusion could have been further developed. Overall, this is a nice descriptive paper.

Response: As mentioned above the recommended vector competence studies are outside the scope of this manuscript, as our intention is to couple them with BSQV pathogenesis in mice and subject to future publication. The discussion and conclusion sections are now further developed with inclusion of caveats of: (i) cell susceptibility does not equal transmission potential, (ii) need for *in vivo* studies to investigate potential sustained transmission and not just spillover, and (iii) strain transmission differences.

**Reviewer #2**

**Methods:**
Reviewer #2: Methods were well described and suitable for the aims/objectives of this study.

Response: Thank you.

**Results:**
Reviewer #2: Results are clear. Only issue was the long section describing each protein. It would be more apprpriate to focus on what was different about the viruses they characterised.

Response: We reduced the length of this section to focus on common/divergent annotation features (architecture, post-translational modifications, sequence) between Bussuquara strains, with fewer mentions of other orthoflaviviruses except defining features (e.g., clade-associated replicate numbers of DB and/or SL structures) and differences (e.g., predicted NS5 N-linked glycosylation). We also retained mention of any PTM sites associated with viral function for other orthoflaviviruses (e.g., predicted NS1 phosphorylation site matching DENV PTM critical for propagation) if they also appeared in BSQV PTM predictions.

**Conclusions:**
Reviewer #2: Conclusions are well supported by their findings.

Response: Thank you.

**Editorial and Data Presentation Modifications?**
Reviewer #2: Minor edits

13. Line 118 replace x with symbol for multiplication.

Response: Corrected as suggested.

14. Line 120. Replace u with greek symbol for micro.

Response: Corrected as suggested.

15. Line 129. Unclear what is meant by next day.

Response: Amended for clarity.

16. Line 136 to 137. Sentence can be simplified.

Response: Amended as suggested.

17. Lines 159 to 164. Results presented in the methods section. Would suggest moving.

Response: Corrected as suggested.

18. Line 222. Suggest "confidence in branching pattern" be replaced by confidence in nodal support.

Response: Corrected as suggested.

19. Line 244. use capital L for litre (like elsewhere).

Response: Corrected as suggested.

20. Figure 1 legend. There are no parentheses in the figure.

Response: Corrected as suggested.

21. Line 392 to 393. Please revise sentence.

Response: Corrected as suggested.

**Summary and General Comments:**
Reviewer #2: This study has characterised historical BSQV isolates from two hosts from a single location and assessed their potential to infect insect and vertebrate cell lines. I think it is important to note that the authors have made use of approaches that do not require animal studies. Their findings are still relevant and address the aims and outcomes outlined in the manuscript. They confirm three were BSQV-like based on nucleotide identity, virion morphology and cytopathology on several cell lines. Characterisation of their DNA and proteins further adds to the conserved features of orthoflaviviruses and differences in cytopathology between two strains will be valuable information about these strains for future studies. The paper highlights a neglected area of research and would be of interest to those unravelling the importance of insect viruses and vector-borne pathogens of humans and other vertebrates.

Major comments
1. Supplementary tables (including all worksheets) require proper headers that describe their contents.

Response: The headers for the supplementary tables are listed in Appendix A. We are also included these headers to their corresponding tables.

2. I couldn't access the zenodo link to assess the raw data.

Response: The zenodo data upload is under embargo until publication and will be available to the reviewer upon request.

3. The manuscript was quite long and the description of each protein-coding region could be significantly summarised to highlight only what was different in the three strains characerised herein.

Response: We reduced the length of this section to focus on common/divergent annotation features (architecture, post-translational modifications, sequence) between Bussuquara strains, with less mentions of other orthoflaviviruses except regarding major differences.

4. Line 142-144. Was the RNA DNase treated? Would this be required for RT step?

Response: The RNA was not DNase treated. Viral stocks were initially clarified by centrifugation to limit cellular debris, and after chloroform separation, collection of the DNA phase was carefully avoided. Quality assessment of extracted RNA indicated minimal genomic DNA contamination.

5. Line 154. Provide a list of taxa/protein domains in the custom database in a supplementary table.

Response: The custom database included all viral protein sequences archived in the GenBank database at the time of analysis (n=10587590 sequences). This statement was clarified in the section. A supplementary table is thus unwarranted and unreasonable given the magnitude of sequences.

6. Line 156 to 158. Add an explanation for the need to align with BWA and bowtie2?

Response: Corrected as suggested.

7. Lines 206 to 209 these are results (move down?)

Response: This paragraph was moved to the section between Genome Characterization and Phylogenetic Analysis results.

8. The BLAST results in Table 2 report the % identity in the local alignment. It should be clear that some regions of the sequences may not have aligned locally and were not included in this result (when compared to a global alignment reported by BLAT, for example).

Response: Table 3 (incorrectly numbered at submission) does not report % identity results from BLAST. We used ClustalW multiple sequence alignment on MacVector software to produce the matrices for both % identity and % similarity of nucleotide and amino acid sequences. The table header and genome characterization results were reworded to clarify the source of calculations.

10. It appears that BeAn 4073 and 4116 were from the same animal and likely represent the same strain? Would it be more appropriate to say that this study characterized two strains only?

Response: So far there is not sufficient evidence to claim these two strains are the same. The records provided by The World Reference Center of Emerging Viruses and Arboviruses do state that BeAn 4116 was isolated from a second blood draw of the same sentinel monkey as the prototype BeAn 4073. The passage histories show a higher number of suckling mouse passages for BeAn 4073 (n=7) versus 4116 (n=1), thus the potential for murine host adaptation cannot be ignored. The strains are homologous at the 5’ and 3’UTR with identical (predicted) folding patterns; however, there is divergence at four amino acids within the E protein and it is unknown if these mutations affect infection phenotype. The *in vitro* infection kinetic curves demonstrated significant albeit low magnitude differences in some cell lines (ex. U4.4). Our ongoing *in vivo* studies in mice and mosquitoes utilize all three strains, and will thus better address this concern. This point has been included the conclusion section to clarify the matching strain origins.

11. Line 363. Here you state that folding differs, but Fig 4 structures look identical apart from some nucleotide differences in blue. Should be clear if differences are with folded templates not shown in the figure.

Response: The beginning of the genome annotation section states that the two strains BeAn 4073 and BeAn 4116 are 100% identical at the nucleotide level within the 5’ and 3’ UTR and thus are represented by the same RNA structure in Figures 3 and 4. There is one predicted folding difference in the 3’UTR (Figure 4) between BeAn 217201 versus BeAn 4116/4073 structures. We revised the description to better specify the extract structural element, preceding both the sHP and 3’SL, containing the divergent residue position correlated to the folding change.

12. Line 395. You mention post-translational modifications, but you are also showing SNPs that confer a synonymous or non-synonymous change. This should be clear to the reader. Also Table 3 header need to be more descriptive, including that these are all predictions (see methods and discussion).

Response: We have now added the word “Predicted” in the table 3 title description. The header itself makes it clear that these are putative sites based on various prediction software.
R**eviewer #3**

**Methods:**
Reviewer #3: Study design and methods are appropriate and adequately described.

Response: Thank you.

**Results:**
Reviewer #3: The experimental results are presented and analyzed in an appropriate manner.

Response: Thank you.

**Conclusions:**
Reviewer #3: The conclusions as currently stated are not adequately supported by the data presented. Limitations are noted but should be more clearly described.

**Editorial and Data Presentation Modifications?**
Reviewer #3: The manuscript should be revised to more clearly focus on the novel observations and findings.

Response: To address this point, we modified the genome annotation section by: (i) removing extraneous details of orthoflavivirus biology (e.g. pseudoknots, sfRNAs), and (ii) removing mentions of other orthoflavivirus post-translational modifications absent in BSQV, while retaining discussion if BSQV had a matched residue PTM to any previously deemed critical for another orthoflavivirus (e.g., NS1 phosphorylation site essential for DENV propagation). Otherwise, we kept details of important structural and sequence motifs, alongside (simplified) explanations of each proteins function.

**Summary and General Comments:**
Reviewer #3: Steck et al report the results of sequencing and in vitro infection using the prototype strain of Bussuquara virus (BSQV, member of Orthoflavivirus aroaense) available through ATCC and the three other strains in the arbovirus reference collection at UTMB. Full genome sequencing was performed and results were annotated and used to determine phylogenetic relationships and to predict RNA secondary structure and protein post-translational modifications. In vitro infection was performed in 7 mosquito cell lines and 10 vertebrate cell lines for measurement of extracellular virus and electron microscopic observations were performed in C6/36 and Vero cells. The main findings were that two of the isolates/strains in the reference collection were confirmed as BSQV whereas the third was identified genetically as Naranjal virus, a related member of Orthoflavivirus aroaense. The three strains of BSQV showed typical genetic characteristics of orthoflaviviruses, induced typical ultrastructural changes in C6/36 and Vero cells, and replicated in most of the mosquito cell lines tested and all of the vertebrate cell lines tested, with minor differences in virus yield between strains but more significant differences in replication between cell lines. The authors main conclusions are that BSQV has genetic characteristics typical of orthoflaviviruses and in vitro replication characteristics that indicate the potential for more extensive epizootic and urban transmission.

The study provides novel information about a poorly characterized arbovirus. The results are generally presented in an appropriate fashion. Given the limited information available on BSQV, the information is likely to be of interest to readers of PLoS Negl Trop Dis. Although the conclusions are largely reasonable, the presentation of the data and conclusions should be improved to better inform the readership.

Specific major comments:

1. Through the manuscript, the authors should conform to the current ICTV nomenclature on BSQV and related members of Orthoflavivirus aroaense. Although the authors acknowledge the current ICTV species designation of the Aroa serocomplex (line 283), the manuscript does not note that all the viruses studied have this same ICTV designation, and NAJV is incorrectly identified as a related species in the Aroa serocomplex (line 267).

Response: Sentence modified to describe NAJV as another member of the Aroa virus group (*Orthoflavivirus aroaense*).

2. Key statements in the Title and Abstract are not adequately supported by the research study, specifically that the work provides "clarification of emergence potential" (Title) and that BSQV "has the capacity to enter epizootic and urban transmission cycles" (Abstract). These should be revised to more accurately reflect the work presented.

Response: The phrase “clarification of emergence potential” was removed from the title. The conclusion that BSQV “has the capacity to enter epizootic and urban transmission cycles” was modified to state that while we conclude BSQV is a generalist arbovirus based on broad vertebrate and mosquito cell susceptibility, we cannot yet make conclusions about urban transmission capacity without supportive *in vivo* studies.

3. The finding of BSQV replication in most of the cell lines tested appears to be the basis for the authors’ conclusions regarding its emergence potential. However, the study does not present justification for this conclusion. In addition to tempering these conclusions in the Title and Abstract as noted above (see #2), the authors should discuss the evidence for and against this interpretation.

Response; We have modified the title, abstract, and discussion section to temper claims of emergence potential, particularly since *in vivo* vector competence studies are pertinent to assess the equivocal susceptibility patterns associated with mosquito genus and immune deficiency.

4. The authors should extend the discussion of prior literature on BSQV (genome annotation and in vitro replication) to the other members of Orthoflavivirus aroaense.

Response: There is substantially less field or experimental data available for other members of the Aroa virus group and thus drawing parallels does not provide elucidation on our findings here. BSQV is the only member of the four associated with human disease. Experimental literature of AROAV, IGUV, and NAJV is primarily associated with pan-orthoflavivirus bioinformatic analyses of UTR RNA structures. Such information is not discussed in the genome annotation as we recognize these are only predictions and because we are not attempting to correlate structure to phenotype at this time.

5. The manuscript should more clearly focus on the novel findings of this study. For example, given the availability of a full genome sequence of BSQV, description of typical genetic features of orthoflaviviruses can be significantly truncated (lines 320-504).

Response: This section is now reduced in size to eliminate extraneous details including pseudoknots, subgenomic RNA, protein functions dependent on cellular location/oligomer state of protein, critical PTMs of other orthoflaviviruses not predicted to be present in BSQV. We focused on major differences (ex. predicted NS5 glycosylation).

6. Lines 530-531- Although statistically significant associations were found, the magnitudes of the differences in viral yield between strains were small; this should be noted.

Response: This point is now added to the results section. We also added details of the largest magnitude difference to support the statement with “albeit the magnitude of differences in viral titer were small (max difference = 1.63 log_10_ PFU/ml, 2dpi FRhL-2).

Specific minor comments:

7. The authors should reiterate in the paragraph on limitations (lines 564-566) that two of the strains studied were isolated from same infected host, thus narrowing further the depth of sampling of BSQV.

Response: This point is now discussed as a major caveat in the conclusion section.

8. The authors should note the # of cell lines tested in the Abstract.

Response: Corrected as suggested.

9. Table 1- It would be helpful to provide the common species names. Tissue culture supplements for CxTr cells should be provided in the table, as is done for the other cell lines.

Response: Table 1 has now been updated to include a column with the common names for each species, and we retained the citation for CxTr cell line as is. Note that the supplements and/or concentrations for this cell line are more specialized compared to the other cell lines and thus omitted from the table (see recipe below). We directly communicated with the lab that established this line to ensure purchase of certain reagents with the same catalog numbers [e.g. "insect tested" FBS - Sigma (F4135)], and thus we felt that guiding researchers to the original publication would allow greater reproducibility.

20% "insect tested" FBS

Schneider's Drosophila Medium

0.4g/L Sodium bicarbonate

0.0585 g/L L-glutamine

0.006 g/L Reduced Glutathione

0.03 g/L L-Asparagine

200 ug/mL Gentamycin

400ug/mL Streptomycin

400U/mL Penicillin

25ug/mL Ciprofloxacin hydrochloride

5ug/mL Amphotericin B

10. Lines 117-119- The authors should note the titers of the viral stocks.

Response: The requested information has now been included.

11. Table 2 (line 123)- It would be helpful to provide the common species names. "SM" should be defined.

Response: Updated as suggested

12. Line 154- Additional details should be provided on custom dataset(s).

Response: The custom database is all viral protein sequences archived in the GenBank database at the time of analysis (n=10587590 sequences). This description is updated in the methods section.

13. Table 2 (line 275; this table is misnumbered)- For the amino acid analysis, the authors should clarify the meaning of values above and below the diagonal (as these are not identical).

Response: Thank you for catching the misnumbered Table 3. The table header was modified to clarify the matrix values as “Similarity scores (relatedness or matches with gaps) are shown below the diagonal with identity scores (exact matches) above as determined with ClustalW multiple sequence alignment.”

14. Figure 2- It is not clear how the authors determined that the virions were immature.

Response: These virions are described as immature due to their noted location in the endoplasmic reticulum. Per the orthoflavivirus virion maturation process, virion maturation majorly occurs during transport to the cell periphery via the trans-golgi network.

15. Lines 534, 538- What is referenced as "intra-strain" differences (versus "inter-strain")?

Response: We have now updated the text for clarity and describe more concisely as “strain” differences.

**Reviewer #4**

**Methods:**
Reviewer #4: (No Response)

**Results:**
Reviewer #4: (No Response)

**Conclusions:**
Reviewer #4: (No Response)

**Editorial and Data Presentation Modifications?**
Reviewer #4: (No Response)

**Summary and General Comments:**
Reviewer #4: Steck et al. present a comprehensive characterization of three historical Bussuquara virus isolates (BeAn 217201, BeAn 4116, and BeAn 4073). The study includes a detailed genome characterization, phylogenetic analysis within the Orthoflavivirus genus, and in vitro replication kinetics in a representative set of vertebrate (including four human) and mosquito cell lines. The methods used are well-described and appropriate and the results are clearly presented. The authors combine the results and discussion sections, which works well especially for the genome characterization and direct comparison with other Orthoflavivirus genomes. The paper also contains a clear discussion of the study’s limitations (only three strains from the same geographic region analyzed...). My main critique of this otherwise solid study concerns the title: the claim that the work “clarifies Bussuquara virus emergence potential” feels somewhat overstated and goes beyond what the presented data fully support, particularly in the absence of in vivo data. I recommend either revising the title or more convincingly addressing this claim in the Discussion

Response: Thank you for the valuable feedback. We agree that without supportive evidence from *in vivo* studies, it is premature to state any clarification of emergence potential. Our ongoing studies are utilizing both mosquitoes and mice. Although these data are outside the scope of this manuscript, they will help to further elucidate the trends of BSQV replication kinetics that we observed across mosquito and vertebrate cell lines. The title was revised by removing “clarifying its emergence potential”.
